# Inner Ear Anatomy Variations in Acute Low-Tone Sensorineural Hearing Loss and Unilateral Stage I/II Ménière’s Disease: A Comparative Study

**DOI:** 10.3390/diagnostics16030473

**Published:** 2026-02-03

**Authors:** Qin Liu, Xingqian Shen, Linlin Wang, Yangming Leng, Cen Chen, Ping Lei, Bo Liu

**Affiliations:** 1 Department of Otorhinolaryngology-Head and Neck Surgery, ENT Institute, Union Hospital, Tongji Medical College, Huazhong University of Science and Technology, Wuhan 430022, China; liuqinent@163.com (Q.L.); sxq15207158114@163.com (X.S.); 2020xh5020@hust.edu.cn (L.W.); lyangming@foxmail.com (Y.L.); 2 Hubei Province Clinical Research Center for Deafness and Vertigo, Wuhan 430022, China; 3 Department of Radiology, Union Hospital, Tongji Medical College, Huazhong University of Science and Technology, Wuhan 430022, China; april_chencen@hotmail.com; 4 Hubei Provincial Clinical Research Center for Precision Radiology & Interventional Medicine, Wuhan 430022, China

**Keywords:** acute low-tone sensorineural hearing loss, Ménière’s disease, endolymphatic hydrops, inner ear anatomical variations, radiology, magnetic resonance imaging

## Abstract

**Objectives**: To investigate the differences in inner ear anatomical variations between patients with acute low-tone sensorineural hearing loss (ALHL) and those with unilateral stage I/II Ménière’s disease (MD) based on magnetic resonance imaging (MRI). **Methods**: A total of 30 patients with unilateral ALHL, 41 patients with unilateral stage I/II MD, and 59 healthy controls were enrolled retrospectively. 3.0T MRI was used to evaluate the distance between the vertical part of the posterior semicircular canal and the posterior fossa (PPD) and vestibular aqueduct (VA) visibility. Inter-group and intra-group comparisons and correlation analyses were performed to clarify the characteristics of anatomical variations. **Results**: (1) There were no significant differences in PPD and VA visibility between ALHL patients and healthy controls; the PPD of unaffected ears in MD patients was significantly shorter than that in healthy controls, while no significant difference was observed in the PPD of affected ears between MD patients and healthy controls. (2) The VA visibility of affected ears in ALHL patients was significantly higher than that in MD patients. (3) No significant intra-group differences in PPD and VA visibility between affected and unaffected ears were noted in ALHL or MD patients. (4) A significant negative correlation was found between the PPD of affected ears and pure tone average of affected ears in MD patients, while no correlations were observed between anatomical indices and clinical characteristics in ALHL patients. **Conclusions**: Although both ALHL and MD are categorized as hydropic ear diseases, radiological evidences demonstrate that MD patients exhibit inner ear anatomical variations, whereas no significant anatomical variations are observed in ALHL patients. This suggests that anatomical variations in the endolymphatic drainage system may be a predisposing factor for the pathogenesis of unilateral MD rather than for unilateral ALHL.

## 1. Introduction

Acute low-tone sensorineural hearing loss (ALHL) is an otological disorder primarily characterized by sudden-onset low-frequency sensorineural hearing loss. Its main clinical manifestations include aural fullness, tinnitus, and hearing loss, with no accompanying vertigo [1]. The pathophysiological mechanism of ALHL remains unclear, and potential mechanisms include endolymphatic hydrops (ELH) and immune responses [2,3]. Previous studies have shown the presence of relevant manifestations of ELH in patients with ALHL during audiological examinations and imaging studies [2,4,5].

Ménière’s disease (MD) is an otological syndrome characterized primarily by recurrent vertigo, fluctuating sensorineural hearing loss, tinnitus, and aural fullness [6]. Patients with stage I/II MD exhibit audiometric curves that may resemble those of patients with ALHL. Both groups present with rising-type curves, characterized by low-frequency hearing loss, and frequently report aural fullness and tinnitus. These shared features contribute to overlapping clinical manifestations between ALHL and stage I/II MD. In addition, clinical observations have shown that symptoms in some ALHL patients may resolve spontaneously, while others may progress to typical MD. Currently, the mainstream view holds that ALHL is an independent disease; however, some studies suggest that ALHL may represent an early stage of MD and that ALHL accompanied by symptoms such as dizziness, tinnitus, and aural fullness is more likely to progress to MD [7].

The pathological basis of MD is ELH, but its specific pathogenesis has not been fully elucidated. It is believed that the development of ELH in patients with MD involves multiple factors, including an imbalance between the production and absorption of endolymph, abnormal ion homeostasis, genetic susceptibility, viral infection, autoimmune response, vascular factors, and vestibular migraine [8]. Anatomical variations in the endolymphatic duct–endolymphatic sac (ED–ES) system in patients with MD have been investigated and proposed as potential contributors to the development of ELH [9]. Imaging and histopathological evidence demonstrate that the ES and vestibular aqueduct (VA) are narrower in MD patients than in healthy individuals [10,11]. In addition, subsequently published imaging studies have further identified multiple anatomical variations in the inner ear of MD patients, including reduced distance between the vertical part of the posterior semicircular canal and the posterior fossa (PPD) [10], poor visualization of the ED or VA [10,12,13], jugular bulb abnormalities [14,15], and abnormal VA morphology [16]. Gluth et al. proposed that MD can be considered part of the spectrum of hydropic inner ear disease, which also includes some disorders without vertigo (such as ALHL) [17]. This perspective not only explains the presence of ELH observed in other diseases but also provides new insights into exploring the ELH-related anatomical characteristics in other disorders. However, there is a paucity of studies investigating the inner ear anatomical characteristics in ALHL patients. In particular, no clear conclusion has been reached regarding whether anatomical abnormalities are associated with the development of ELH in ALHL patients.

To address this research gap, the present study retrospectively analyzed the MRI findings of patients with unilateral ALHL and those with unilateral stage I/II MD, aiming to investigate the differences in MRI-visualized anatomical variations in the inner ear between these two diseases and provide anatomical evidence and theoretical support for the pathophysiological mechanism of related diseases in the future. Clinically, differentiating ALHL from first-onset or early-stage MD can be difficult at the initial visit, due to the absence of reliable markers. At the same time, ALHL remains under-investigated at the anatomical level, and the potential progression to MD has not been thoroughly explored. This constitutes an important scientific and clinical knowledge gap. Investigating anatomical similarities and differences between ALHL and MD may ultimately enable the development of more robust diagnostic tools and predictive models. Future integration of imaging, audio-vestibular function, and clinical parameters may allow estimation of the likelihood of ALHL progressing to MD.

## 2. Materials and Methods

### 2.1. Participants

This study was conducted at Union Hospital, Tongji Medical College, Huazhong University of Science and Technology, where a total of 30 patients with unilateral ALHL, 41 patients with unilateral stage I/II MD, and 59 healthy control subjects were enrolled.

The diagnosis of ALHL was based on the diagnostic criteria revised by the Ministry of Health, Labour and Welfare of Japan in 2024 [1]. This diagnostic criterion includes the following key symptoms: (1) acute or sudden-onset cochlear symptoms, including aural fullness, tinnitus, and hearing loss; (2) low-frequency hearing loss; (3) absence of vertigo; (4) unknown etiology. Reference indicators include the following: (1) audiological criteria for low-frequency hearing loss: ① the sum of hearing thresholds at low frequencies (0.125, 0.25, and 0.5 kHz) ≥ 70 dB and ② the sum of hearing thresholds at high frequencies (2, 4, and 8 kHz) ≤ 60 dB; (2) recurrent cochlear symptoms; (3) potential progression to MD; (4) possible accompanied by mild dizziness; (5) bilateral onset possible. A confirmed diagnosis requires meeting all key symptoms and both audiological criteria ① and ②. The diagnosis of MD was based on the diagnostic criteria proposed by the Bárány Society in 2015 [18]. The diagnostic criteria include the following key symptoms: (1) two or more spontaneous episodes of vertigo, each lasting 20 min to 12 h; (2) audiometrically documented low to medium frequency sensorineural hearing loss in one ear, defining the affected ear on at least one occasion before, during, or after one of the episodes of vertigo; (3) fluctuating aural symptoms (hearing, tinnitus, or fullness) in the affected ear; (4) not better accounted for by another vestibular diagnosis. Patients with stage I/II MD were enrolled in this study, with the mean hearing threshold at three frequencies (500, 1000, and 2000 Hz) ≤ 40 dB. All patients underwent detailed medical history collection, otoscopic examination, audio-vestibular function assessment, and imaging studies. The exclusion criteria were as follows: (1) middle or inner ear infection; (2) history of ear surgery or intratympanic injection; (3) retrocochlear lesions; (4) bilateral MD or bilateral ALHL, or abnormal hearing thresholds in the unaffected ear; (5) history of head trauma; (6) central nervous system diseases; (7) concomitant vestibular disorders: VM, benign paroxysmal positional vertigo, and related conditions; (8) systemic diseases, such as autoimmune disorders, cardiovascular diseases, or metabolic syndromes.

This study was conducted in compliance with the latest version of the Declaration of Helsinki. This retrospective study was approved by the Ethics Committee of Union Hospital, Tongji Medical College, Huazhong University of Science and Technology on 1 June 2025, and the requirement for informed consent from participants was waived (Institutional Review Board approval [2025] No. 0506).

### 2.2. Methods

#### 2.2.1. Audio-Vestibular Function Assessment

In the present study, all MD patients underwent audio-vestibular function assessment during the interictal period. All subjects were instructed to avoid alcohol, caffeine, and medications (such as sedatives, antidepressants, diuretics, carbonic anhydrase inhibitors, and betahistine) that might affect the test results within 48 h before vestibular function testing.

Pure tone audiometry was performed using a standard audiometer. Hearing thresholds at frequencies of 0.125, 0.25, 0.5, 1, 2, 4, and 8 kHz were recorded. The pure tone average (PTA) at three frequencies (500, 1000, and 2000 Hz) was calculated to evaluate the degree of hearing loss.

#### 2.2.2. Imaging Methods

All participants underwent MRI examinations using a Siemens Verio or Magnetom Trio 3 T scanner (equipped with a 12-element phased-array coil), with T1-weighted and T2-weighted spin-echo imaging techniques employed (key parameters: T1-weighted: Repetition Time (TR) = 300 ms, Echo Time (TE) = 2 ms, slice thickness = 5 mm, and Field of View (FOV) = 250 mm × 250 mm; T2-weighted: TR = 9000 ms, TE = 90 ms, slice thickness = 5 mm, and FOV = 220 mm × 200 mm). Additionally, three-dimensional sampling perfection with application-optimized contrasts using different flip-angle evolutions (3D-SPACE) (key parameters: TR = 1000 ms, TE = 135 ms, slice thickness = 0.5 mm, and FOV = 200 mm × 200 mm) was used to quantify the distance between the vertical part of the posterior semicircular canal and the posterior fossa and VA visibility.

**Figure 1:**
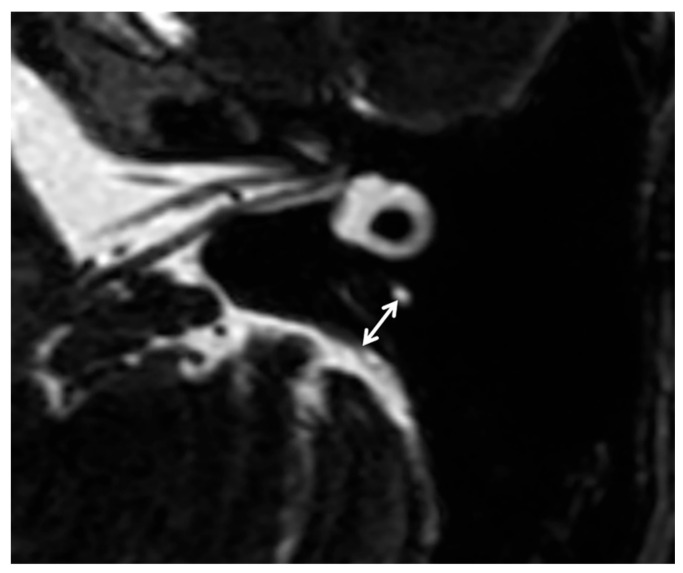
Axial 3D-SPACE MRI scan showing a detailed image of the left ear at the level of the measured distance between the vertical part of the posterior semicircular canal and the posterior fossa.

**Figure 2:**
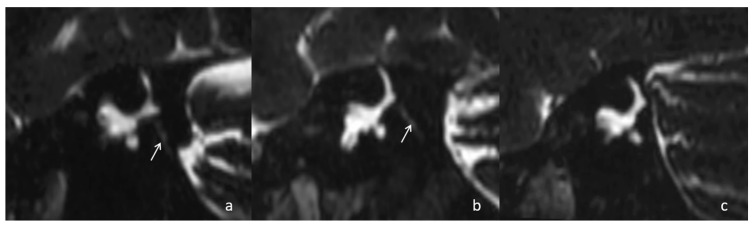
VA scoring on Pöschl views of 3D-SPACE images: (**a**) grade 0—continuous visualization of the entire VA (arrow); (**b**) grade 1—discontinuous visualization of the VA (arrow); (**c**) grade 2—no visualization of the VA.

The anatomical variations included in MRI-visualized measurements in this study were the distance between the vertical part of the posterior semicircular canal and the posterior fossa visualized by MRI (PPD) and the VA visibility. The PPD was measured on 3D-SPACE axial images, which were parallel to the bilateral horizontal semicircular canal (Figure 1) [10]. The VA visibility was also measured on 3D-SPACE axial images, which refers to the continuous display of a linear or dot-like high signal intensity along the direction from the common crus to the posterior edge of the temporal bone on multiple MRI sections, and it is classified into three grades: grade 0 denotes continuous visualization of the entire VA; grade 1 indicates discontinuous visualization of the VA; grade 2 represents no visualization of the VA (Figure 2) [19].

All MRI data were transferred to workstations, and image analysis was performed on a picture archiving and communication system (PACS) workstation (Carestream Client, Carestream Health). Two senior radiologists with experience in otological image interpretation mixed the radiological data of all subjects, then conducted a blinded review of the clinical data. All images with interpretive discrepancies were discussed by these two radiologists to reach a consensus.

#### 2.2.3. Statistical Analyses

SPSS 25.0 software (SPSS Inc., Chicago, IL, USA) was utilized for processing clinical data and conducting statistical analyses. The Shapiro–Wilk test was used to assess the normality of the measurement data, while the Levene test evaluated the homogeneity of variance for the normally distributed data. For measurement data that followed a normal distribution, results were presented as $\bar{x}$ ± s, and a *t*-test was employed. In contrast, measurement data that did not conform to the normal distribution were summarized as M (P25, P75), and a non-parametric test was applied. The count data were displayed by frequency, and the chi-square test was used for analysis.

For correlation analyses, the appropriate correlation coefficient (Pearson’s correlation coefficient for normally distributed variables or Spearman’s rank correlation coefficient for non-normally distributed variables) was selected based on the distribution characteristics of the data. Within-group comparisons were performed between the affected and unaffected ears of the same patients using paired statistical tests, whereas between-group comparisons were conducted across different groups using analyses of covariance (ANCOVAs). Furthermore, ANCOVAs were performed with age and sex as covariates to control for potential confounding effects.

## 3. Results

### 3.1. Demographic and Clinical Features

The demographic and clinical features of patients with unilateral ALHL and patients with unilateral stage I/II MD are presented in Table 1. A total of 30 patients were enrolled in the ALHL group, including 24 females (80.0%) and 6 males (20.0%), with a mean age of 30.50 ± 8.81 years; pure tone average (PTA) of the affected ear in this group was 20.50 (17.29, 23.80) dB HL, and the PTA of the unaffected ear was 12.22 ± 3.31 dB HL. In the MD group, 41 patients were enrolled, including 21 females (58.5%) and 17 males (41.5%), with a mean age of 45.24 ± 11.99 years; the PTA of the affected ear in this group was 29.67 (24.80, 37.10) dB HL, and the PTA of the unaffected ear was 13.90 ± 6.07 dB HL. The healthy control group consisted of 40 females and 19 males, with a median age of 54.40 (38.10, 60.13) years.

### 3.2. Radiological Evaluations

#### 3.2.1. Comparisons of the PPD

**Figure 3:**
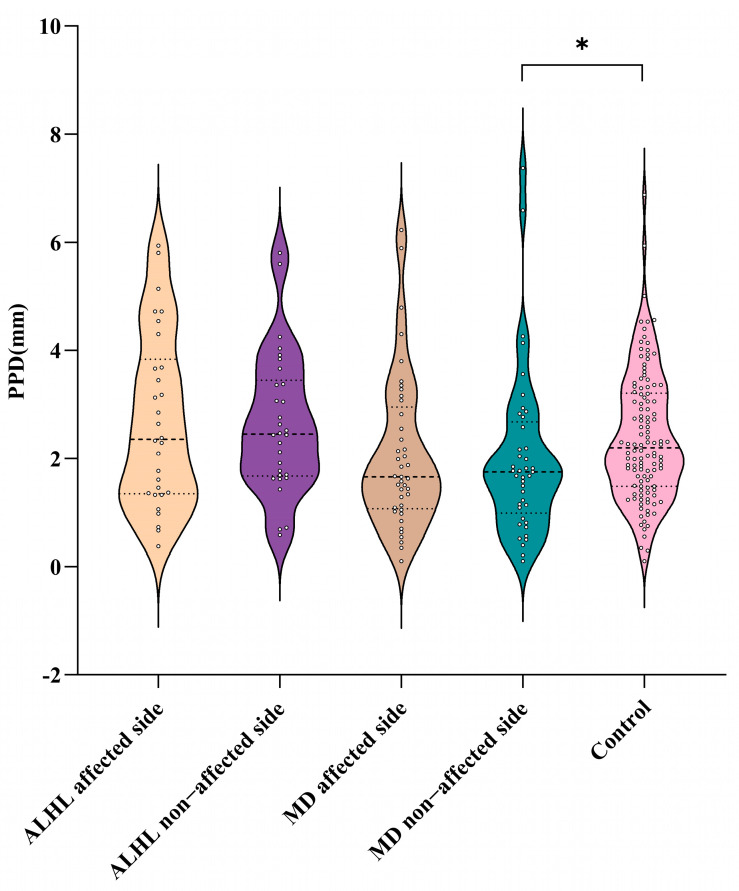
Comparisons of the PPD. ALHL: acute low-tone sensorineural hearing loss; MD: Ménière’s disease; PPD: distance between the vertical part of the posterior semicircular canal and the posterior fossa visualized by MRI. *: *p* < 0.05.

As shown in Table 2 and Figure 3, among 30 patients with unilateral ALHL, the PPD of the affected ear was 2.70 ± 1.60 mm and that of the unaffected ear was 2.63 ± 1.29 mm. Among 41 patients with unilateral stage I/II MD, the median PPDs of the affected and unaffected ears were 1.66 (1.07, 2.95) mm and 1.75 (0.99, 2.68) mm. For healthy control subjects, the mean PPD of the left ear was 2.38 ± 1.27 mm and the median PPD of the right ear was 2.15 (1.67, 3.19) mm, with no statistically significant difference in PPD between the left and right ears (Z = −0.694, *p* = 0.487). The left and right ears of healthy control subjects were combined into one group (118 ears in total), with a median PPD of 2.20 (1.49, 3.21) mm.

Additionally, there was no statistically significant difference in PPD between the affected ears of ALHL patients and those of MD patients (F = 0.321, *p* = 0.573). For affected ears, there was no statistically significant difference in PPD between the ALHL patients and the healthy control subjects (F = 0.281, *p* = 0.597) and no statistically significant difference in PPD between the MD patients and the healthy control subjects (F = 3.641, *p* = 0.058). For unaffected ears, there was also no statistically significant difference in PPD between the ALHL patients and the healthy control subjects (F = 0.594, *p* = 0.442), but the PPD of MD patients was shorter than the healthy control subjects (F = 4.935, *p* = 0.028). No statistically significant difference in PPD was noted between the unaffected and affected ears of ALHL patients (t = −0.392, *p* = 0.698), and the same was true for MD patients (Z = −0.484, *p* = 0.628).

Although no statistically significant difference was observed in the PPD of the affected ear in MD patients compared with healthy controls, it showed a trend toward being smaller.

#### 3.2.2. Comparisons of the VA Visibility

**Figure 4:**
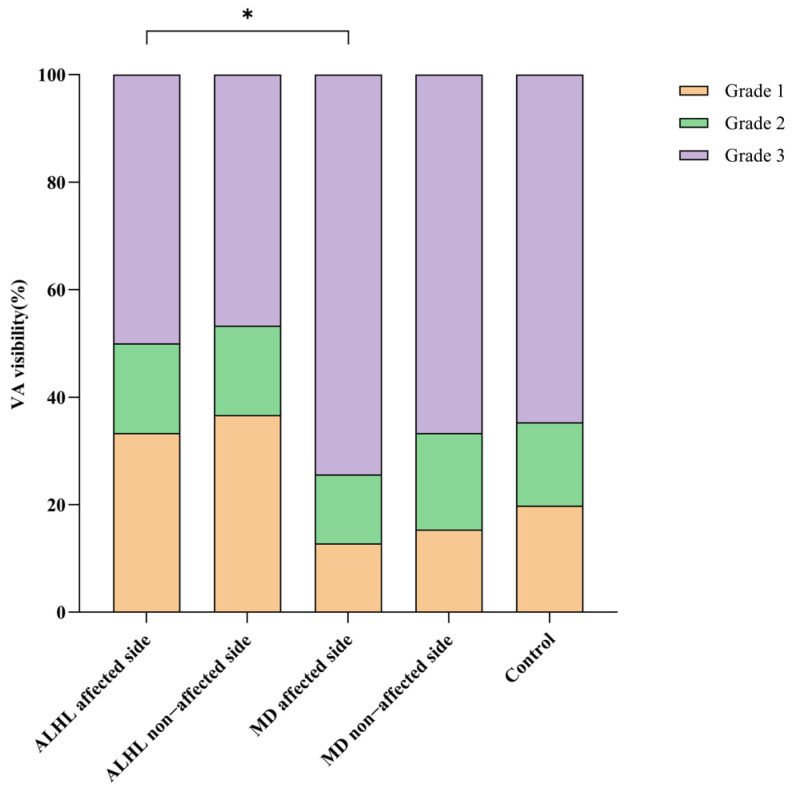
Comparisons of VA visibility. ALHL: acute low-tone sensorineural hearing loss; MD: Ménière’s disease; VA: vestibular aqueduct. *: *p* < 0.05.

As shown in Table 3 and Figure 4, for patients with ALHL, the percentage of grade 0 VA visibility in the affected ear was 33.3% (10/30), grade 1 was 16.7% (5/30), and grade 2 was 50% (15/30); in the unaffected ear, grade 0 accounted for 36.7% (11/30), grade 1 was 16.7% (5/30), and grade 2 was 46.7% (14/30). Among 41 patients with unilateral stage I/II MD, two cases had missing VA visibility data, and a total of 39 patients were finally included for analysis; in this group, the percentage of grade 0 VA visibility in the affected ear was 12.8% (5/39), grade 1 was 12.8% (5/39), and grade 2 was 74.4% (29/39). In contrast, in the unaffected ear, grade 0 accounted for 15.4% (6/39), grade 1 was 17.9% (7/39), and grade 2 was 66.7% (26/39). Among 59 healthy control subjects, one case had missing VA visibility data, and 58 subjects were finally included; the grading percentages of VA visibility in their left and right ears were as follows: left ear: grade 0 was 25.9% (15/58), grade 1 was 10.3% (6/58), and grade 2 was 63.8% (37/58); right ear: grade 0 was 13.8% (8/58), grade 1 was 20.7% (12/58), and grade 2 was 65.5% (38/58). No statistically significant difference in VA visibility was observed between the left and right ears of healthy control subjects (Z = −1.007, *p* = 0.314). When the left and right ears of healthy control subjects were combined for analysis (116 ears in total), the grading percentages of VA visibility were as follows: grade 0 was 19.8% (23/116), grade 1 was 15.5% (18/116), and grade 2 was 64.7% (75/116).

Additionally, compared with the affected ears of MD patients, the affected ears of ALHL patients had higher VA visibility (F = 4.678, *p* = 0.034). For affected ears, there was no statistically significant difference in VA visibility between the ALHL patients and that of healthy control subjects (F = 0.266, *p* = 0.607) and no statistically significant difference in VA visibility between the MD patients and the healthy control subjects (F = 1.995, *p* = 0.160). For unaffected ears, there was also no statistically significant difference in VA visibility between the ALHL patients and the healthy control subjects (F = 0.674, *p* = 0.413) and no statistically significant difference in VA visibility between the MD patients and the healthy control subjects (F = 0.631, *p* = 0.428). No statistically significant difference in VA visibility was noted between the unaffected and affected ears of ALHL patients (Z = −0.513, *p* = 0.608), and the same was true for MD patients (Z = −0.973, *p* = 0.331).

### 3.3. Correlation Between Anatomical Indices and Clinical Features in ALHL and MD Patients

**Figure 5:**
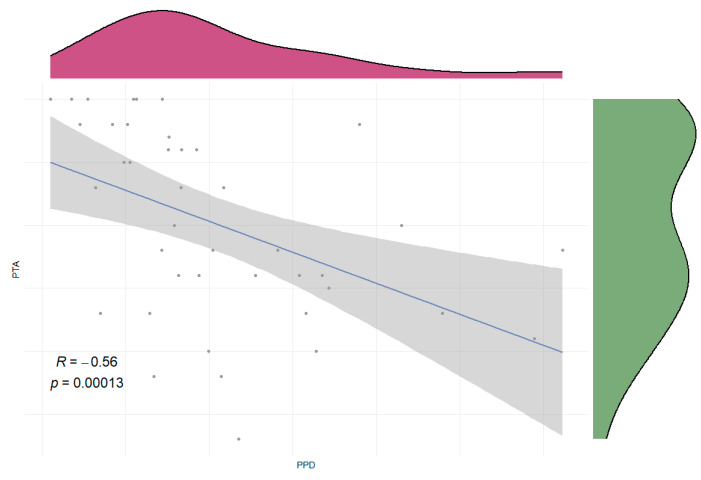
Correlation between the PPD and PTA in the affected ear of patients with MD. PPD: distance between the vertical part of the posterior semicircular canal and the posterior fossa visualized by MRI; The green density plot on the right displays the data distribution of PTA, and the red density plot at the top illustrates the data distribution of PPD; PTA: pure tone average; MD: Ménière’s disease.

In this study, correlation analyses were performed between anatomical characteristics and clinical characteristics in ALHL patients and MD patients separately. Among these, anatomical characteristics included the PPD and the VA visibility, and clinical characteristics included age, gender, affected side, and the pure tone average (PTA) of the unaffected and affected ears. None of the correlations in ALHL patients showed statistical significance (all *p* > 0.05). However, in MD patients, the PPD of the affected ear exhibited a significant negative correlation with the PTA of the affected ear (R = −0.56, *p* < 0.001) (Figure 5).

A significant negative correlation was observed between the PPD of the affected ear and the PTA of the affected ear in patients with unilateral stage I/II MD (R = −0.56, *p* < 0.001). In contrast, no statistically significant correlations were found between anatomical characteristics (PPD and VA visibility) and clinical factors (age, gender, affected ear side, and PTA of both ears) in patients with unilateral ALHL (all *p* > 0.05).

## 4. Discussion

### 4.1. Differences in Anatomical Characteristics Between ALHL and MD Patients

In the present study, there was no difference in PPD between ALHL patients and healthy control subjects. Compared with healthy control subjects, MD patients had a shorter PPD in the unaffected ear. To a certain extent, the length of the PPD can reflect the volume of the ES. Albers et al. [10] and Bernard et al. [20] found that the PPD was shortened in the affected ear or both ears of patients with unilateral MD, which is consistent with the result of the present study. In fact, although no statistically significant change in the PPD of the affected ear was observed in MD patients in this study, the indicator still showed a decreasing trend. Notably, Oya et al. found that there was no significant change in the PPD in MD patients [21], which suggests that future studies need to further explore the anatomical characteristics of MD patients.

To date, the etiology and pathogenesis of ALHL have not been fully elucidated [2], and potential contributing factors may include ELH [5], immune factors [22,23], and migraine [24]. ECochG and vestibular evoked myogenic potentials (VEMPs) have been used for the functional assessment of ELH. Noguchi et al. [4] and Jiang et al. [25] found that some ALHL patients exhibit abnormal ECochG and VEMP findings, suggesting that ELH may be present in ALHL. And Seo et al. confirmed the presence of ELH in ALHL patients via gadolinium-enhanced MRI [2]. All these studies suggest that ALHL may share a common pathophysiological basis with MD, namely ELH. At present, the majority of scholars consider ALHL a distinct disease entity, whereas others propose that ALHL represents an early stage of MD. According to published research reports, 9–17% of patients with ALHL may progress to MD [3,26,27]. Due to the lack of longitudinal observation in the present study, the included ALHL patients who potentially progress to MD may have attenuated the anatomical differences between MD patients and ALHL patients who did not eventually progress to MD.

In the present study, there was no difference in VA visibility between MD patients and healthy controls; similarly, no difference was observed in VA visibility between ALHL patients and healthy controls. The VA is a bony canal which encloses the ED and the intraosseous portion of the ES. It is generally accepted that the VA visibility reflects the patency of the ED. Sugiura et al. demonstrated that compared with controls without hearing loss, there was no difference in the ED visualization rate in both ears of ALHL patients, which was similar to our findings [28]. However, the comparison results between patients with MD and healthy subjects are inconsistent with previous research [10,13,16,19,29], which may be attributed to differences in the imaging technique, judgment criteria, and grading systems for VA/ED visibility. Furthermore, the results of the present study still show a decreasing trend in VA visibility in the affected ears of patients with MD, which is similar with the findings of previous studies [10,13,16,19,29].

This study found that the affected ears of ALHL patients had higher VA visibility than the affected ears of MD patients. de Pont et al. demonstrated that non-visualization of the ED-ES system is more prevalent in patients with definite MD [9]. Similar findings have also been reported by Gerb et al. [30] and Attyé et al. [19], which further supports the conclusions of the present study. In addition, Huang et al. proposed that the morphological characteristics of the VA are associated with the occurrence and development of MD as well as the degree of ELH [16]. Although numerous imaging studies have shown ELH features in both ALHL and MD, the differential VA visibility demonstrated in this study may indicate that anatomical factors contribute more substantially to MD than to ALHL. This suggests that the decreased visibility of VA may play a more important role in MD rather than in ALHL and that the pathogenesis of ALHL may differ from that of MD.

### 4.2. Differences in Anatomical Characteristics Between the Affected and Non-Affected Sides

In the present study, there were no differences in PPD and VA visibility between affected and unaffected ears in patients with ALHL and those with MD, respectively. Albers et al. [10], Lorenzi et al. [13], and Mateijsen et al. [31] found that there was no significant difference in PPD between affected and unaffected ears in patients with unilateral MD. Attyé et al. found no significant difference in bilateral MRI-VA visibility in patients with unilateral MD [19]. In contrast, Huang et al. investigated bilateral VA visibility in patients with unilateral MD using three-dimensional real inversion recovery (3D-real IR) sequences and found that the no-visualization rate of the VA was significantly higher in the affected ear (91.1%) than in the unaffected ear (41.1%) [32]. These discrepancies may be explained by differences in imaging technology and VA visibility grading approaches. Recently, based on studies about ATVA, Bächinger et al. found that unilateral MD patients with bilateral ES hypoplasia are at risk of bilateral disease progression [33]. Therefore, although our results did not identify anatomical differences between the affected and unaffected ears in either MD or ALHL, determining whether the disease course involves bilateral involvement still requires long-term follow-up incorporating additional imaging parameters.

### 4.3. Correlation Differences in Anatomical Characteristics and PTA

The correlation analysis in this study revealed differences in the association between anatomical characteristics and hearing loss severity (measured by PTA) between ALHL and MD.

In MD patients, a shorter PPD of the affected ear was associated with a higher PTA, which may be attributed to the “functional reserve” hypothesis [34]. As the core structure responsible for endolymph absorption, anatomical abnormalities of the ES directly impair endolymph absorption function. Specifically, a shorter PPD indicates a smaller ES volume and lower functional reserve, resulting in weaker compensatory capacity for endolymph metabolic disorders and subsequently more severe ELH [34]. Severe ELH damages the normal physiological functions of cochlear hair cells and auditory nerve fibers, ultimately manifesting as aggravated hearing loss. The hearing of MD patients is fluctuating and progressive. Therefore, the impact of different anatomical characteristics on hearing progression in MD patients requires further studies.

The lack of an association between anatomical characteristics and PTA in ALHL patients may be attributed to the unknown etiology of ELH in ALHL. We speculate that ELH in ALHL may be unrelated to anatomical factors. Existing studies have shown that ELH in ALHL may be associated with other factors such as abnormal drainage of the vascular plexus around the ED, ion channel dysfunction, and vasopressin imbalance [35,36,37], rather than congenital anatomical variations in the ES/ED. Yoon et al. found that small vessel disease may be an underlying cause of sudden sensorineural hearing loss and may also be associated with its recurrence [38]. However, whether vascular factors are associated with anatomically mediated impairment of the endolymphatic drainage system remains unclear and warrants further investigation. Therefore, even if ELH is present in some ALHL patients, their hearing loss is mainly driven by other factors.

## 5. Strengths and Limitations

The present study focuses on ALHL and unilateral stage I/II MD, two hydropic ear diseases with overlapping clinical phenotypes and similar rising-type audiometric curves. However, their pathological mechanisms remain poorly differentiated. By specifically examining differences in inner ear anatomical variations between these two conditions, this study effectively fills the research gap regarding the anatomical mechanisms of ALHL.

Admittedly, the present study has certain limitations. Firstly, during the diagnosis of ALHL and MD patients, this study did not routinely perform gadolinium-enhanced inner ear MRI, which could visualize ELH in vivo. Secondly, in the present study, there were no differences in PPD and VA visibility between patients with unilateral stage I/II MD and healthy controls. This result is not entirely inconsistent with previous studies and may be related to the relatively small sample size. To further explore the role of anatomical variations in the pathophysiology of hydropic ear diseases, additional prospective controlled studies with a larger sample are required in the future. Thirdly, some ALHL patients may progress to MD, and our study is a retrospective cross-sectional study with a lack of longitudinal follow-up observations. The long-term observations may be more beneficial for understanding the impact of anatomical variations on the pathophysiology and prognosis of ALHL.

## 6. Conclusions

Although ALHL and MD are both categorized as hydropic ear diseases, the present study demonstrates that MD patients exhibit congenital inner ear anatomical variations, whereas no statistically significant anatomical variations are observed in ALHL patients. This suggests that anatomical changes in the endolymphatic drainage system may be a predisposing factor for the pathogenesis of unilateral MD rather than for unilateral ALHL.

## Figures and Tables

**Table 1:** The demographic and clinical features of patients with ALHL and MD.

	ALHL	MD	HC
age (years)	30.50 ± 8.81	45.24 ± 11.99	54.40 (38.10, 60.13)
gender (male/female)	6/24	17/24	19/40
affected ear (left/right)	17/13	26/15	/
PTA of affected ears (dB HL)	20.50 (17.29, 23.80)	29.67 (24.80, 37.10)	/
PTA of non-affected ears (dB HL)	12.22 ± 3.31	13.90 ± 6.07	/

Note: ALHL: acute low-tone sensorineural hearing loss; MD: Ménière’s disease; PTA: pure tone average; HC: healthy control. The PTA at three frequencies (500, 1000, and 2000 Hz) was calculated.

**Table 2:** The PPD of patients with ALHL, MD and healthy control subjects.

	PPD (mm)
ALHL affected ear	2.70 ± 1.60
ALHL unaffected ear	2.63 ± 1.29
MD affected ear	1.66 (1.07, 2.95)
MD unaffected ear	1.75 (0.99, 2.68)
healthy control	2.20 (1.49, 3.21)

Note: ALHL: acute low-tone sensorineural hearing loss; MD: Ménière’s disease; PPD: distance between the vertical part of the posterior semicircular canal and the posterior fossa visualized by MRI.

**Table 3:** The VA visibility of patients with ALHL, MD and healthy control subjects.

	VA Visibility		
	Grade 0	Grade 1	Grade 2
ALHL affected ear	10 (33.3%)	5 (16.7%)	15 (50.0%)
ALHL unaffected ear	11 (36.7%)	5 (16.7%)	14 (46.7%)
MD affected ear	5 (12.8%)	5 (12.8%)	29 (74.4%)
MD unaffected ear	6 (15.4%)	7 (17.9%)	26 (66.7%)
healthy control	23 (19.8%)	18 (15.5%)	75 (64.7%)

Note: ALHL: acute low-tone sensorineural hearing loss; MD: Ménière’s disease; VA: vestibular aqueduct.

## Data Availability

The data presented in this study are available on request from the corresponding authors. The data are not publicly available due to privacy or ethical restrictions.

## Abbreviations

The following abbreviations are used in this manuscript:

ALHL	Acute low-tone sensorineural hearing loss
MD	Ménière’s disease
MRI	Magnetic resonance imaging
PPD	The distance between the vertical part of the posterior semicircular canal and the posterior fossa
VA	Vestibular aqueduct
ES	Endolymphatic sac
ELH	Endolymphatic hydrops
ED	Endolymphatic duct
ECochG	Electrocochleogram
PTA	Pure tone average
VEMP	Vestibular evoked myogenic potential
cVEMP	Cervical vestibular evoked myogenic potential
oVEMP	Ocular vestibular evoked myogenic potential
ATVA	Angular trajectory of the vestibular aqueduct
ΔpSCC-post	The sagittal distance between the most posterior border of the posterior semicircular canal and the posterior temporal bone surface
